# Serotonergic Neuromodulation of Natural Products Isoreserpine and Isoreserpiline in Adult Zebrafish: An in Silico and In Vivo Investigation

**DOI:** 10.1002/cbdv.202501738

**Published:** 2025-11-21

**Authors:** Nádia Aguiar Portela Pinheiro, Ivana Carneiro Romão, Amanda Maria Barros Alves, Sônia Maria Costa Siqueira, Jane Eire Silva Alencar de Menezes, Emmanuel Silva Marinho, Maria Kueirislene Amâncio Ferreira, Márcia Machado Marinho, Herbert de Sousa Magalhães, Andreia Ferreira de Castro Gomes, Otília Deusdênia Loiola Pessoa, Hélcio Silva dos Santos

**Affiliations:** ^1^ Graduate Program in Natural Sciences State University of Ceará Fortaleza Brazil; ^2^ Center For Exact Sciences and Technology Vale Do Acaraú State University Sobral Brazil; ^3^ Department of Organic and Inorganic Chemistry Federal University of Ceará Fortaleza Brazil; ^4^ Environmental Biology, Department of Biology School of Sciences, University of Minho Braga Portugal

**Keywords:** anxiety, DMPK, indole alkaloids, light/dark test, molecular docking

## Abstract

Anxiety is a multidimensional behavioral disorder widely studied in neuroscience due to the involvement of specific neural circuits. Although benzodiazepines are commonly used, their side effects drive the search for new therapeutic alternatives. This study investigated the anxiolytic effects of the indole alkaloids isoreserpine (IRPN) and isoreserpiline (IRPL), extracted from *Rauvolfia ligustrina*, in adult zebrafish at doses of 4, 12, and 20 mg/kg, along with 3% DMSO and diazepam (4 mg/kg) as controls. Toxicity, locomotor activity, anxiolytic effects, and serotonergic involvement were assessed. Interactions with the 5‐HT3AR receptor were analyzed through molecular docking, and pharmacokinetic properties were evaluated using DMPK. The samples showed no toxicity (LD_50_ > 20 mg/kg), reduced locomotor activity, and exhibited anxiolytic effects that were reversed by granisetron. IRPL demonstrated higher affinity for the 5‐HT3AR receptor and better absorption and central nervous system penetration properties, standing out as a promising candidate for the treatment of anxiety.

## Graphical abstract

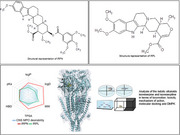

## 1 Introduction

The genus *Rauvolfia*, popularly known as devil's pepper, belongs to the Apocynaceae family and is represented by 74 species distributed across tropical countries worldwide, including *Rauvolfia tetraphylla*, *Rauvolfia serpentina*, *Rauvolfia ligustrina*, among others [1, 2]. For a long time, studies on species of this genus have focused on the isolation and characterization of compounds with potential biological activities, as well as investigating the presence of indole alkaloids such as sarpagine, ajmaline, reserpine, serpentine, reserpiline, aricine, IRPL, yohimbine, and IRPN, which have been isolated from different species of the genus [2]. The presence of indole alkaloids is associated with medicinal properties, and many pharmacological studies have been reported in the literature, including anti‐inflammatory, antipsychotic, cardioprotective, anxiolytic, sedative activities, among others [1, 2].

*R. ligustrina* has been investigated for its anticonvulsant potential [3] and effects on noradrenergic neurotransmission [4]. There are also reports related to anxiolytic activity investigated by Mendonça Netto et al. [5], who evaluated the ethanolic extract of R. ligustrina roots, and Sousa et al. [6], who also used the ethanolic extract but isolated several indole alkaloids and concluded that sarpagine, rauvovertine B, perakisine, and sarpaginine showed good anxiolytic results.

**FIGURE 1:**
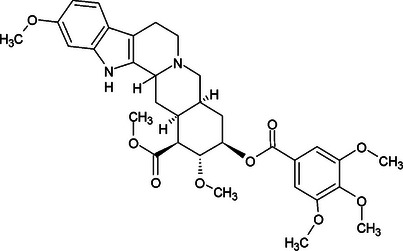
Structural representation of IRPN.

**FIGURE 2:**
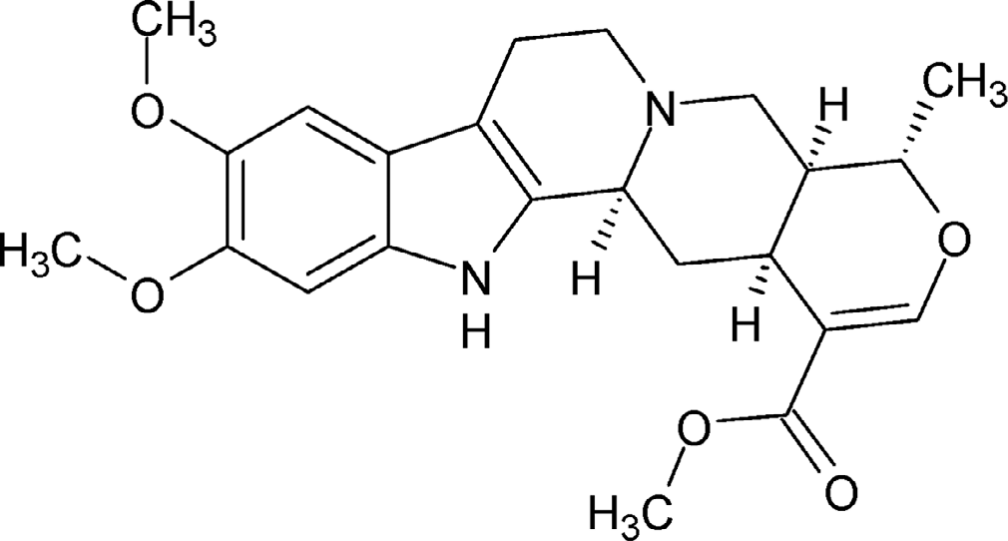
Structural representation of IRPL.

Despite research on the *Rauvolfia* genus, some indole alkaloids, such as IRPN (Figure 1) and IRPL (Figure 2), which have already been isolated from several species, including *R. ligustrina*, have not yet been investigated regarding their anxiolytic effects [6]. The literature reports studies involving antipsychotic [7, 8], antibacterial [9, 10], anticholinesterase [11], and anticancer [12] activities for IRPL. For IRPN, only anticancer activity studies have been found [13, 14].

Considering the significant increase in anxiety cases worldwide—with about 1 billion people suffering from mental disorders—and the side effects associated with prolonged benzodiazepine use, such as dependence, excessive drowsiness, cognitive impairment, and risk of falls, it becomes evident that there is a need to develop anxiolytic drugs with minimized side effects [15, 16].

Therefore, this study aimed to evaluate the toxicity and anxiolytic activity of IRPN and IRPL, obtained from *R. ligustrina*, using zebrafish as an animal model. In addition, pharmacodynamic (molecular docking) and pharmacokinetic (ADMET) analyses of the compounds were carried out to better understand their therapeutic potential.

## 2 Results and Discussion

### 2.1 Acute Toxicity

The IRPN and IRPL samples were evaluated for acute toxicity in adult zebrafish at doses of 4, 12, and 20 mg/kg. Neither of the compounds exhibited significant toxicity, as no mortality rate exceeded 50% at any of the tested concentrations (LD_50_ > 20 mg/kg; Table 1). These results support the continuation of in vivo pharmacological investigations to further assess the efficacy and safety of the tested doses.

**TABLE 1:** Results of acute toxicity tests of IRPN and IRPL in adult zebrafish.

Sample	Mortality				96 h, LD_50_ (mg/kg)/CI
	NC	D1	D2	D3	
IRPN	0	2	1	3	>20
IRPL	0	0	2	0	>20

Abbreviations: CI, confidence interval; D1, Dose 1 (4 mg/kg); D2, Dose 2 (12 mg/kg); D3, Dose 3 (20 mg/kg); LD_50_, lethal dose to kill 50% of adult zebrafish; NC, negative control—DMSO (3%; 20 µL, ip).

The findings of this study are consistent with the data reported by Huang et al. [14], who observed low toxicity of IRPN in studies focused on chemotherapy treatment for cancer. Similarly, Gupta et al. [7] evaluated the toxicity of the methanolic extract from the leaves of *R. tetraphylla* L., which contains IRPL, and found no toxic effects in Swiss albino mice exposed to doses of 10, 100, 300, and 2.000 mg/kg over seven days, supporting the data obtained in this study.

### 2.2 Open Field Test

**FIGURE 3:**
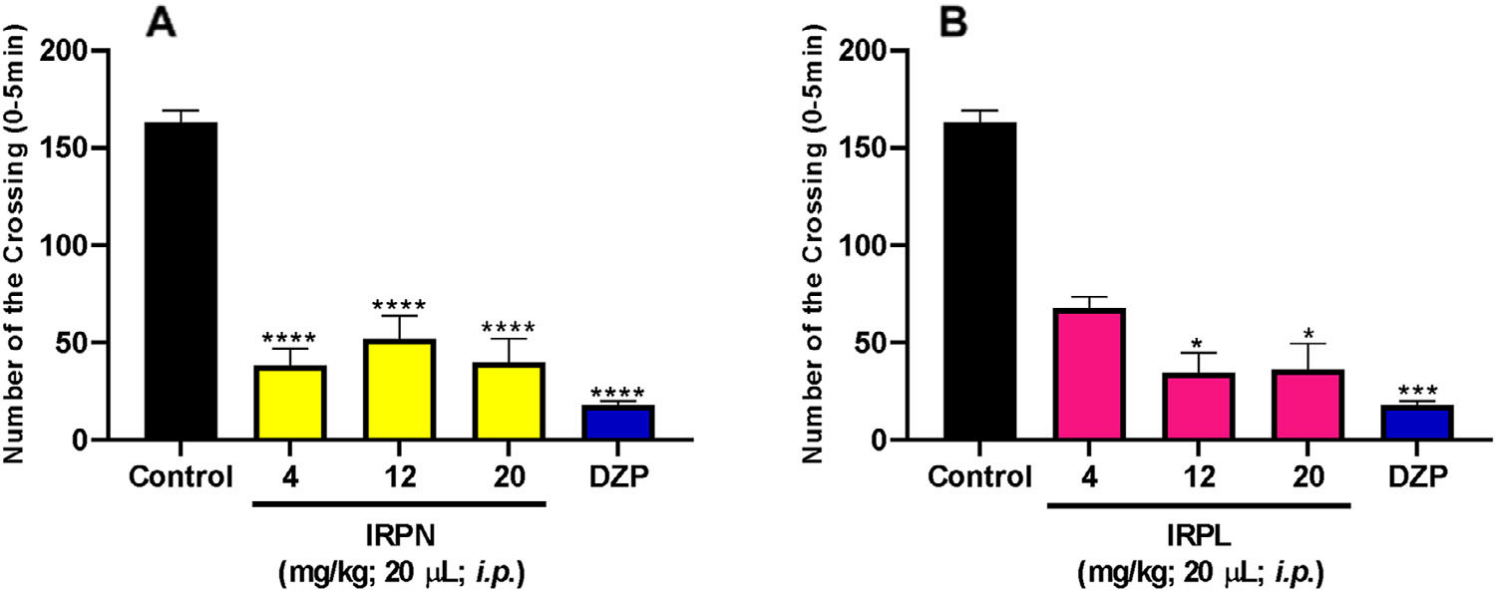
Effect of IRPN and IRPL on zebrafish locomotor activity. DZP—diazepam (4 mg/kg; 20 µL/animal, ip). Control—DMSO (3%; 20 µL, ip). The values represent the mean ± standard error of the mean (SEM) for six animals/group. ANOVA followed by Tukey for the IRPN sample and Kruskal–Wallis test followed by Dunn for the IRPL sample; *p < 0.05; ***p < 0.001; ****p < 0.0001 versus negative control.

The results obtained from the open field test (Figure 3) demonstrated that the alkaloids significantly altered the locomotor activity of zebrafish compared to the negative control. Administration of IRPN resulted in statistically significant differences at all tested doses (****p < 0.0001) (Figure 3A). In contrast, treatment with IRPL showed significant effects only at doses of 12 and 20 mg/kg (*p < 0.05) (Figure 3B), with these effects being comparable to those observed with diazepam (DZP) (***p < 0.001).

Gupta and colleagues [6, 7, 17], reported the isolation of the indole alkaloid IRPN from *R. tetraphylla* L., confirming the presence of this compound in the plant. In contrast, Madawala et al. [18] investigated the sedative activity of the crude extract from the dried and powdered root bark of R. tetraphylla L., observing that doses of 50 and 100 mg/kg (ip) significantly reduced the locomotor activity of male albino rats. According to Mahalakshimi et al. [19], sedative activity appears to be a recurring feature among reserpine‐type alkaloids. Therefore, the data obtained in the present study are in agreement with the literature and further suggest that the reduction in locomotor activity induced by IRPN may occur at lower doses than those previously reported.

In addition, it is important to note that antipsychotic drugs act on the central nervous system (CNS) and often exhibit sedative and psychomotor effects. In this context, Gupta et al. [7] isolated six indole alkaloids, including IRPL, from the methanolic extract of R. tetraphylla L. leaves. The authors reported that administration of IRPL at a dose of 25 mg/kg significantly reduced the motor activity of mice, as measured by the distance traveled over a 5‐min period, when compared to the amphetamine‐treated group. Thus, the sedative effects described in the literature are consistent with the findings of the present study, which demonstrated that IRPL induces sedation at lower doses than those used by Gupta et al. [7].

### 2.3 Anxiolytic Activity

**FIGURE 4:**
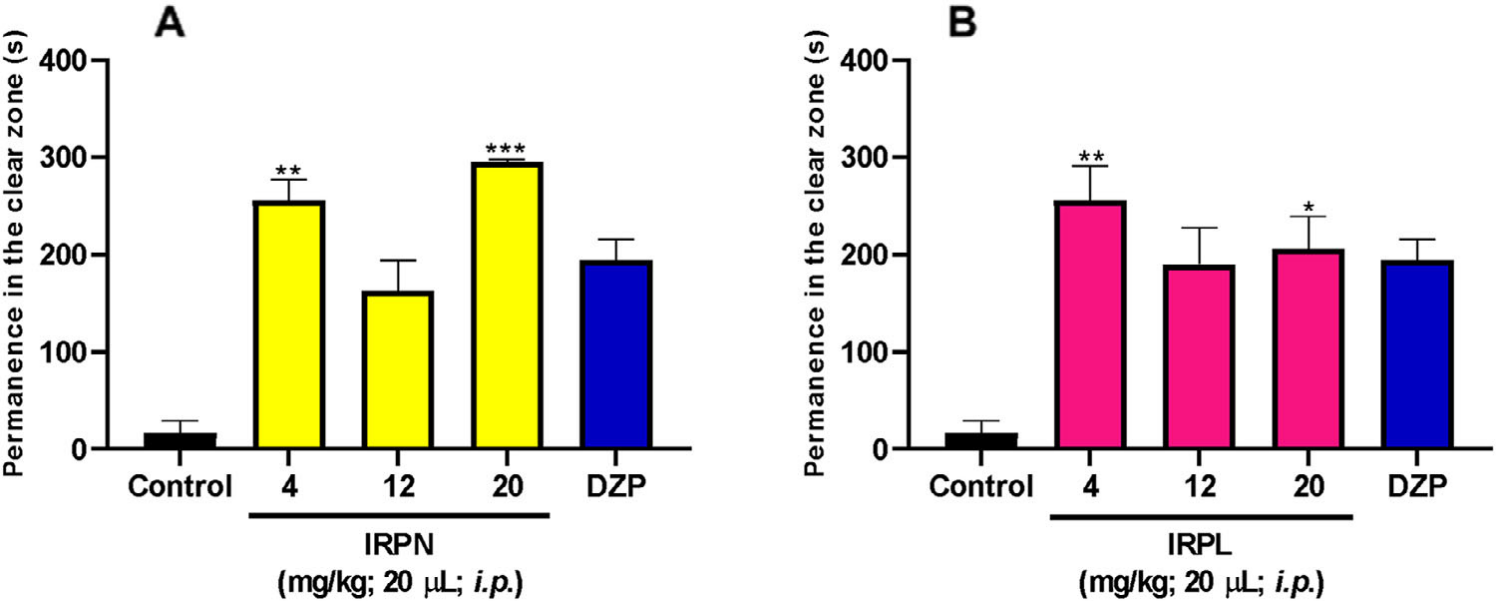
Anxiolytic effect of IRPN and IRPL in adult zebrafish in the light/dark test. DZP—Diazepam (4 mg/kg; 20 µL/animal, ip); negative control—DMSO (3%; 20 µL, ip). The values represent the mean ± standard error of the mean for six animals/group; Kruskal–Wallis test followed by Dunn; *p < 0.05; **p < 0.01; ***p < 0.001 versus negative control.

The results obtained from the light/dark test (Figure 4) indicated that the alkaloids IRPN and IRPL produced anxiolytic effects in adult zebrafish when compared to the negative control, as the animals spent more time in the light zone of the tank (Figure 4). Both compounds showed statistically significant effects at doses of 4 and 20 mg/kg, with IRPN demonstrating highly significant differences (**p < 0.001 and ***p < 0.001 vs. negative control) (Figure 4A), and IRPL showing similar results (**p < 0.01 and *p < 0.05 vs. negative control) (Figure 4B).

Considering that DZP is widely recognized as a reference drug for anxiety treatment, it is noteworthy that the effects induced by IRPN and IRPL were more pronounced than those observed with DZP, which showed only a visual difference compared to the negative control.

Thus, the dose of 4 mg/kg was considered the most promising for both alkaloids, as it produced a significant anxiolytic effect without inducing relevant adverse effects, and was therefore selected for the investigation of the mechanisms of action. The use of lower doses represents an important therapeutic advantage, as it reduces the risk of side effects, lowers toxicity, improves tolerability, and increases clinical safety—factors that enhance the therapeutic viability of these compounds [20].

To date, no studies have been found in the literature that specifically investigate the anxiolytic activity of the alkaloids IRPN and IRPL evaluated in this study. However, Sousa et al. [6] isolated other indole alkaloids from R. ligustrina—such as sarpagine, rauvovertine B, peraksine, and sarpaginine—and assessed their anxiolytic activity using zebrafish as an animal model. The authors reported positive anxiolytic effects for all tested compounds, with 4 mg/kg being the most effective dose. These findings support the results of the present study and reinforce the evidence that indole alkaloids represent a promising class for the development of new drugs for the treatment of anxiety.

### 2.4 Evaluation of Serotonergic Neuromodulation (5‐HT)

**FIGURE 5:**
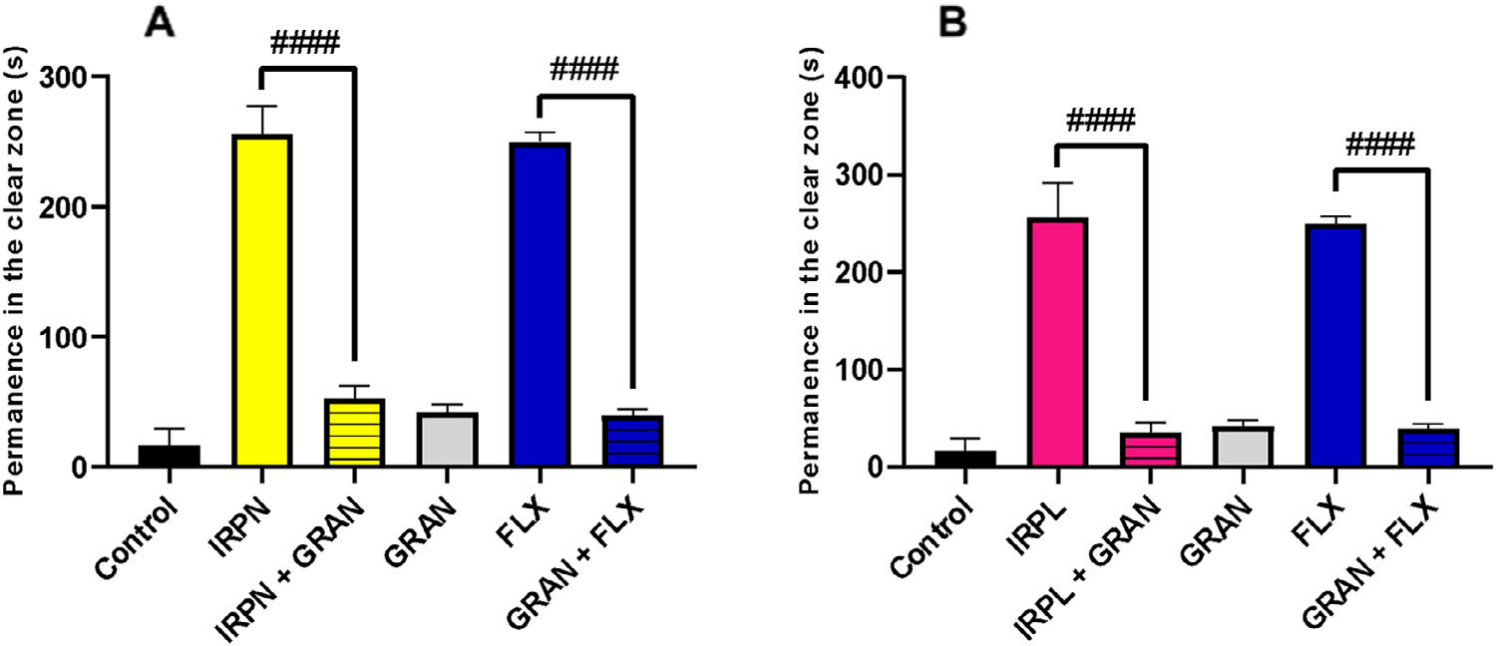
(A) Evaluation of IRPN and its interaction with granisetron (GRAN) and fluoxetine (FLX). ####p < 0.0001 IRPN versus IRPN + GRAN; ####p < 0.0001 FLX versus FLX + GRAN. (B) Evaluation of IRPL and its interaction with GRAN and FLX. ####p < 0.0001 IRPL versus IRPL + GRAN; ####p < 0.0001 FLX versus FLX + GRAN. FLX (0.05 mg/kg; 10 µL/animal, ip); GRAN (20 mg/kg; 10 µL, orally); negative control—DMSO (3%; 20 µL/animal, ip). The values represent the mean ± standard error of the mean for six animals/group; two‐way ANOVA followed by Tukey.

The anxiolytic mechanism of action was investigated through the serotonergic pathway, using the most effective dose identified for the alkaloids (4 mg/kg). The anxiolytic effects induced by IRPN and IRPL were significantly reversed by the administration of the 5‐HT receptor antagonist granisetron (GRAN), as demonstrated by the statistical differences observed between IRPN and IRPN + GRAN (####p < 0.0001) (Figure 5A), and between IRPL and IRPL + GRAN (####p < 0.0001) (Figure 5B). A similar result was observed for FLX, with a significant reversal of its effects (####p < 0.0001 FLX vs. FLX + GRAN), reinforcing the involvement of the serotonergic system in the anxiolytic effect. As a consequence of this reversal, the zebrafish returned to anxious behavior, characterized by increased time spent in the dark zone of the tank.

Sousa et al. [6] investigated the mechanism of action of indole alkaloids—sarpagine, rauvovertine B, peraksine, and sarpaginine—using their most effective anxiolytic doses (4 mg/kg). The authors concluded that sarpagine, rauvovertine B, and peraksine exerted their anxiolytic effects predominantly through the GABAergic pathway, while the anxiolytic effect of sarpaginine was reversed by inhibition of the serotonergic pathway, indicating the involvement of this system in its action.

### 2.5 Molecular Docking

To investigate the potential anxiolytic mechanism of the derivatives IRPN and IRPL, the drug‐target profile associated with the 5‐HT3AR receptor was evaluated through a series of independent molecular docking simulations. At the end of the simulation cycle, an affinity energy value of approximately −8.3 kcal/mol was observed for the IRPN–5‐HT3AR complex and about −6.7 kcal/mol for the IRPL–5‐HT3AR complex, with RMSD values around 1.6 Å, reflecting the quality of the simulations (optimal when < 2.0 Å). This low root mean square deviation (RMSD) indicates good reproducibility of the simulation model (Table 2) [21]. Thus, affinity energy values below the ideal threshold of −6.0 kcal/mol indicate favorable complex formation between the ligands and the 5‐HT3AR receptor (Table 2) [22].

**TABLE 2:** Data from molecular docking simulations of IRPN and IRPL ligands with the 5‐HT3A receptor, including RMSD parameters, affinity energy, and details of ligand–receptor interactions.

Ligand	RMSD (Å)	Energy (kcal/mol)	Interactions	
			Type	Residue (distance in Å)
IRPN	1.606	−8.3	Hydrophobic	Val225 (3.51), Pro230 (3.14), Leu266 (3.42), Ile267 (3.75), Ile267 (3.06), Ala275 (3.27)
			H‐bond	Ser226 (2.56), Ser270 (3.19), Ala275 (2.67), Thr280 (2.43)
IRPL	1.668	−6.7	Hydrophobic	Trp63 (3.80), Thr154 (2.35), Trp156 (3.16), Trp156 (2.84), Trp156 (2.80), Ile201 (3.52)
			H‐bond	Asn101 (2.98), Arg169 (3.23), Arg169 (2.81)
CWB^a^	—	—	Hydrophobic	Ile44 (3.79), Trp63 (3.71), Arg65 (3.76), Trp156 (3.65), Phe199 (3.53), Tyr207 (3.65)
			π‐Cátion	Arg65 (4.12), Arg65 (4.40)

^a^ CWB: Granisetron—antagonist (control) used for comparison in molecular docking simulations.

**FIGURE 6:**
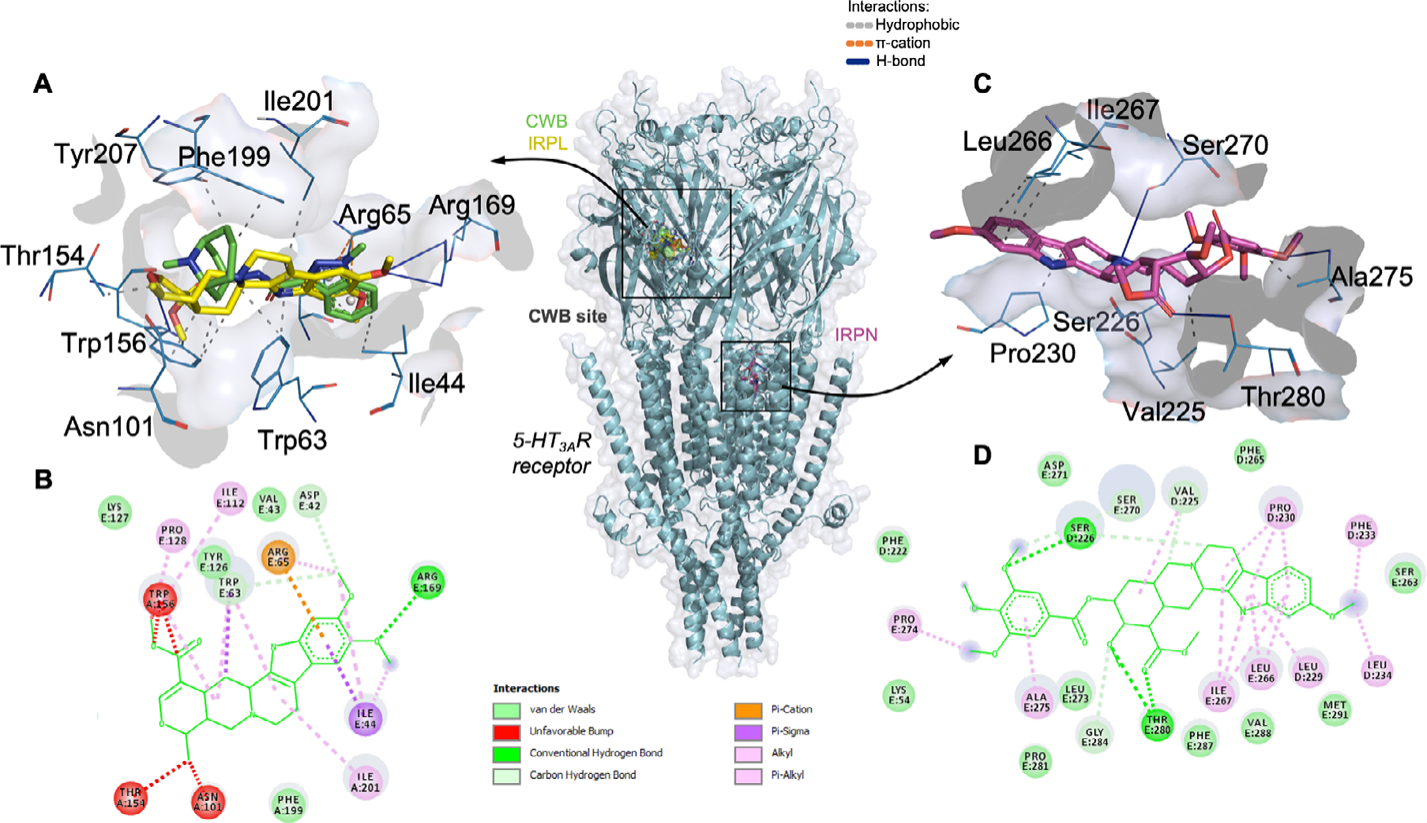
Ligand–receptor interactions of the ligands IRPL (yellow), expressed in (A) three‐dimensional and (B) two‐dimensional forms, and RPN (magenta), expressed in (A) three‐dimensional and (B) two‐dimensional forms, and compared to the antagonist CWB (green) in the 5‐HT3AR receptor (cyan).

When analyzing ligand–receptor interactions, it was observed that the ligand IRPL can bind to the 5‐HT3AR receptor by forming interactions similar to those of the antagonist CWB (Figure 6A), whose binding site is located between chains A and E (Table 2). IRPL formed hydrophobic interactions with the aromatic side chains of residues Trp63 (Chain E) and Trp156 (Chain A), mainly through its fused heterocyclic ring (Figure 6B). In addition, the ligand formed hydrogen bonds with the polar side chains of residues Asn101 (2.98 Å) and Arg169 (2.81 Å), where the ligand–receptor distances below 3.0 Å (Table 2) indicate moderate‐strength hydrogen bonds [23]. Conversely, the ligand IRPN bound to the transmembrane domain of the 5‐HT3AR receptor (Figure 6C), forming hydrophobic interactions with non‐aromatic residues including Val225, Pro230, Leu266, Ile267, and Ala275, as well as hydrogen bonds with the polar side chains of residues Ser226, Ser270, Ala275, and Thr280, located between chains D and E (Figure 6D). The results suggest that IRPL and CWB compete for the same binding site on 5‐HT3AR receptors, corroborating the antagonist's ability to reverse the anxiolytic effect of IRPL in in vivo tests.

With the results obtained, it was observed that the ligand IRPL forms interactions with residues characteristic of the binding site of known 5‐HT3AR antagonists, suggesting that this compound may act as an inhibitor of serotonergic receptors and serve as an alternative anxiolytic [24]. Sousa et al. [6] conducted molecular docking studies on several indole alkaloids such as sarpagine, 3‐hydroxy‐sarpagine, ligustrine, and risperidone. They concluded that the sarpaginic cores act via the GABAergic system, while the others showed affinity for the serotonergic receptor, although both interacted at different sites from the antagonist, as the residues identified by the authors differ from those of CWB described in Table 2. It is worth noting that no molecular docking data were found for IRPN, whereas IRPL's inhibitory activity on cholinesterase has been investigated using this technique [11].

### 2.6 Normal Mode Analysis‐Based Molecular Dynamics Simulation

**FIGURE 7:**
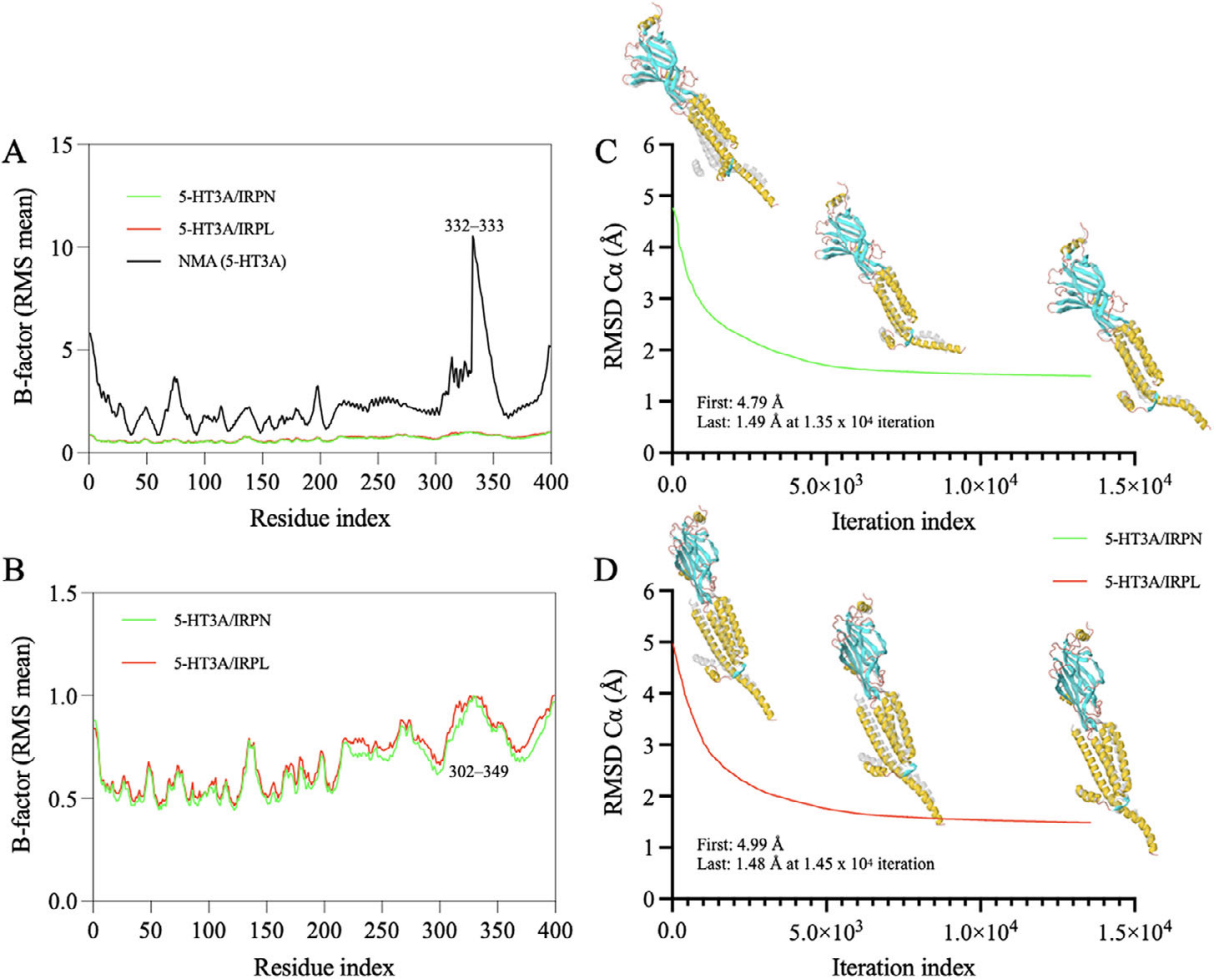
(A) B factors derived from normal mode analysis (NMA) showing intrinsically flexible regions of the 5‐HT3A subunit (black line), highlighting the segment between residues 332–333. (B) RMS mobility profiles per residue (RMS mean) of the 5‐HT3A/IRPN (green) and 5‐HT3A/IRPL (red) complexes, indicating that both ligands stabilize the receptor in a similar manner, with greater local fluctuations concentrated in the distal domain (residues 302–349). (C, D) Convergence of the morphing trajectory for the two complexes, monitored by the RMSD of Cα atoms throughout the iterations. (C) The 5‐HT3A/IRPN complex converged to a final RMSD of 1.49 Å in ∼1.35 × 10^4^ iterations, while the (D) 5‐HT3A/IRPL complex converged to 1.48 Å in ∼1.45 × 10^4^ iterations.

Molecular dynamics (MD) simulations were conducted using the normal mode analysis (NMA) module to observe the deformability of the ligand–receptor complex that the compounds form when binding to the 5‐HT3A receptor. When the NMA module was applied to the PDB of the 5‐HT3A receptor (6np0:A), where GRAN (CWB: black line) is complexed, it was possible to observe a deformability in B factors (RMS mean) around 10 Å between residues 332–333 (Figure 7A). Conversely, a discernible diminution in the flexibility of the residues is evident when the IRPN (green line) and IRPL (red line) compounds are bound to their corresponding residues in the E (6np0:E) and A (6np0:A) chains, respectively. In this case, the RMS mean values are substantially lower than 5.0 Å (Figure 7A).

This behavior is readily discernible in the vicinity of the residue sequence 302–349, which exhibited the most significant RMS Mean values, approaching 1.0 Å for lower values (see Figure 7B). In this NMA trajectory, it is evident that, despite their relative stability in relation to the original PDB, the 5‐HT3A/IRPN complex (green line) exhibited enhanced stability in terms of low conformational flexibility when compared to the 5‐HT3A/IRPL complex (red line). This observation aligns with the energy state of affinity observed in molecular docking simulations, where IRPN can act synergistically with CWB with an affinity energy lower than −8.0 kcal/mol (Table 2). For this simulation, a free binding energy (Δ*G*_binding_) of −5.26 kcal/mol is predicted for the 5‐HT3A/IRPN complex and −5.32 kcal/mol for the 5‐HT3A/IRPL complex. These findings indicate that the complexes exhibit thermodynamically similar stabilities in the NMA module.

Upon analysis of the iteration trajectory, it was ascertained that the NMA mode accentuated the conformational disparities between the GABAA receptor structure at the inception and conclusion of the MD simulations. The outcomes were articulated as a divergence in the Cα portion within the conformational space. In the trajectory of the 5‐HT3A/IRPN complex (green line), it is possible to observe an initial RMSD of approximately 4.79 Å, with convergence iteration of approximately 1.35 × 10^4^ (calculation step), resulting in an RMSD of around 1.4 Å (see Figure 7C). The calculated value between 1.0 and 2.0 Å expresses a plausible physiological movement, without exaggeration or structural collapse [25, 26]. For the 5‐HT3A/IRPL complex (red line), an initial RMSD of approximately 4.99 Å is observed, where the structure stabilizes with an RMSD of 1.48 Å and convergence iteration in the order of 1.45 × 10^4^ (Figure 7D). This indicates that the structure required more iteration cycles to converge when compared to the 5‐HT3A/IRPN complex. The results of the NMA‐based MD simulations suggest that collective movements for both complexes are stable.

### 2.7 In Silico DMPK Studies

#### 2.7.1 CNS Multiparameter Optimization Desirability

It is essential that drug candidates targeting the CNS demonstrate significant therapeutic effects with minimal toxicity. According to Wager et al. [27] and Sun et al. [28], within the CNS Multiparameter Optimization (MPO) framework, compounds with low lipophilicity (log *P* ≤ 3), higher molecular weight (MW 200–500 g/mol), and greater polarity (topological polar surface area [TPSA] between 40 and 90 Å^2^) tend to present a better balance between pharmacokinetic properties—such as passive cell permeability (*P*_app_) and metabolic stability—and safety [23].

**FIGURE 8:**
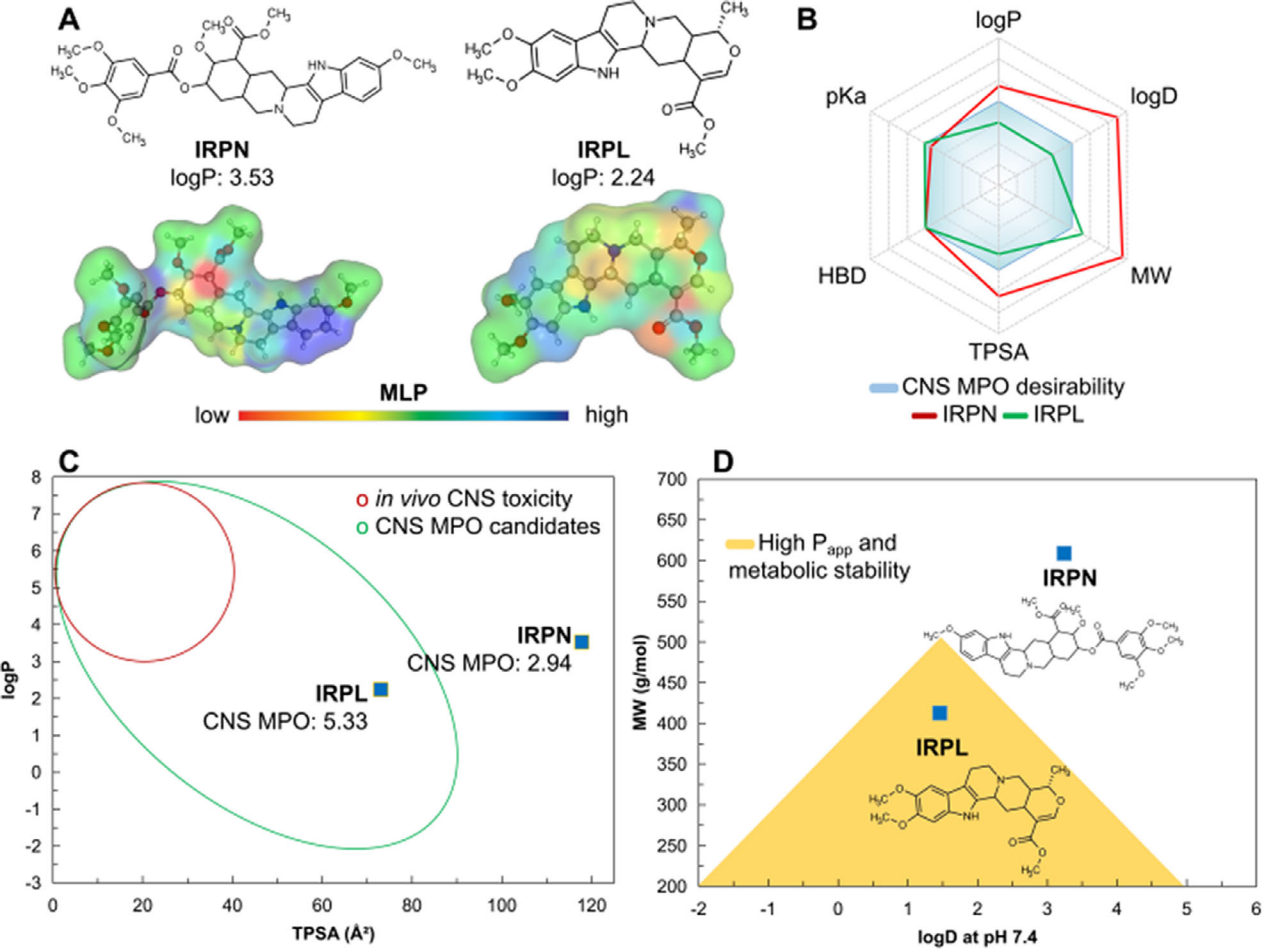
(A) Two‐dimensional representation and molecular lipophilicity potential (MLP) surface of the IRPN and IRPL derivatives, whose physicochemical attributes were applied to the (B) CNS MPO system for drug‐likeness scoring. The MPO descriptors were analyzed for (C) CNS permeation potential, by aligning lipophilicity (log *P*) and polarity (TPSA), and (D) permeability potential (*P*_app_) and metabolic stability, by aligning solubility at physiological pH (log *D* at pH 7.4) and MW.

Through a topological analysis of molecular lipophilicity potential (MLP), it is possible to assess the contribution of molecular fragments to the permeability of small molecules across the cell lipid bilayer [29, 30]. In this analysis (Figure 8A), the indole aromatic moiety represents a highly hydrophobic region (blue color spectrum), while the fused heterocyclic substructure shows greater affinity for polar environments (yellow to red spectrum). In addition, the ─OCH_3_ groups increase the lipophilicity of IRPN (green to blue spectrum) compared to the IRPL analog. On the other hand, IRPL exhibits a better balance between polar surface (NH, N, and O groups) and hydrophobic surface (aromatic ring), indicating a more favorable profile between solubility and lipophilicity—with calculated log *P* values of 2.24 (optimal range: 0–3) for IRPL and 3.53 for IRPN.

The presence of five ─OCH_3_ groups contributed to a polar surface area of 117.78 Å^2^, increasing the lipophilicity (log *P* and log *D*) and molecular weight (MW) of the IRPN derivative. These properties shifted the compound outside the optimal drug‐likeness spectrum defined by the CNS MPO algorithm (Figure 8B), resulting in a CNS MPO score of 2.94 (on a scale from 0 to 6). In contrast, the IRPL derivative exhibited a polar surface area of 73.02 Å^2^, with strong contributions from NH (12.03 Å^2^) and C═O (17.07 Å^2^) groups. This led to a better fit within the drug‐likeness spectrum (Figure 8B) and a higher CNS MPO score of 5.33 (Table 3).

**TABLE 3:** Physicochemical properties of IRPN and IRPL derivatives calculated and applied to the CNS MPO scoring system from Pfizer Inc.

Compound	log *P*	log *D*	MW (g/mol)	TPSA (Å^2^)	HBD	p*K*_a_	CNS MPO
IRPN	3.53	3.24	608.69	117.78	1	7.39	2.94
IRPL	2.24	1.46	412.49	73.02	1	8,09	5.33

Abbreviations: CNS MPO, central nervous system multiparameter optimization; HBD, hydrogen bond donors; log *D*, solubility at physiological pH; log *P*, lipophilicity; MW, molecular weight; TPSA, topological polar surface area.

#### 2.7.2 Parallel Artificial Membrane Permeability Prediction

To assess the drug metabolism and pharmacokinetics (DMPK) profile, parallel artificial membrane permeability (PAMPA) descriptors were predicted and analyzed according to the biopharmaceutical classification system of Pfizer Inc. [31, 32]. The alignment of low lipophilicity (log *P* < 3) and high polarity (TPSA > 40 Å^2^) makes compounds safer for CNS use (Figure 8C), where permeability across the BBB is gradual and minimally effective (indicating low in vivo toxicity). However, the TPSA of 117.78 Å^2^ places IRPN above the ideal threshold for CNS activity, while IRPL lies within an optimized physicochemical space among compounds with CNS activity and low toxic response (Figure 8C).

In addition, a molecular weight above 500 g/mol is another limiting factor for IRPN's pharmacokinetics, whereas IRPL shows a better alignment between permeability and metabolic stability [33], especially due to its low lipophilicity at physiological pH (log D at pH 7.4) of 1.46 and a molecular weight below 450 g/mol (Figure 8D).

These analyses are supported by the estimated PAMPA descriptors, where a Papp (MDCK) value of 5.1 × 10−5 cm/s highlights IRPL as having excellent passive cell permeability. When combined with its low passive efflux probability (Peff), this results in an oral absorption of at least 73.66% (Table 4). This high permeability and low Peff indicate that IRPL can readily cross the BBB, making it a more favorable candidate for anxiolytic action compared to its analog IRPN (Table 4). These findings corroborate the in vivo tests, showing that IRPL increased the time spent in the light zone (TSLZ) of the tested species, showing a higher time ratio compared to IRPN (Figure 5). In addition, the anxiolytic effect of the compound can be reversed by the presence of GRAN (CWB), supporting the hypothesis that IRPL may be a more potent BBB permeant than IRPN. The active profile in the CNS was strongly influenced by the molecular weight and lipophilicity of the compound, which is within the expected threshold for compounds with BBB permeability [34].

**TABLE 4:** DMPK attributes expressed in PAMPA descriptors, predicted for the IRPN and IRPL derivatives using the ADMETlab and ADMETboost tools.

Compound	*P*_app_ MDCK (10^−5^ cm/s)	P‐gp	PAMPA (*P*_eff_)	BBB class	Fa%
IRPN	4.5	Substrate	0.027	CNS−	67.98
IRPL	5.1	No substrate	0.002	CNS+	73.66

Abbreviations: BBB, blood–brain barrier permeability; Fa%, fraction absorbed in the gastrointestinal tract; PAMPA, parallel artificial membrane permeability assay prediction; *P*_app_ MDCK, apparent permeability in Madin‐Darby canine kidney cells; *P*_eff_, efflux potential; P‐gp, P‐glycoprotein substrate.

#### 2.7.3 Metabolic Stability Prediction

Studying the metabolic stability of drug candidates allows the estimation of toxicological risks associated with the presence of reactive metabolic sites. These metabolic processes are primarily mediated by cytochrome P450 (CYP450) isoforms in the human liver microsome (HLM) system (Phase I metabolism), which can generate reactive secondary metabolites capable of binding to nonspecific receptors and DNA, potentially resulting in toxicity at both microsomal and systemic levels [35, 36].

**FIGURE 9:**
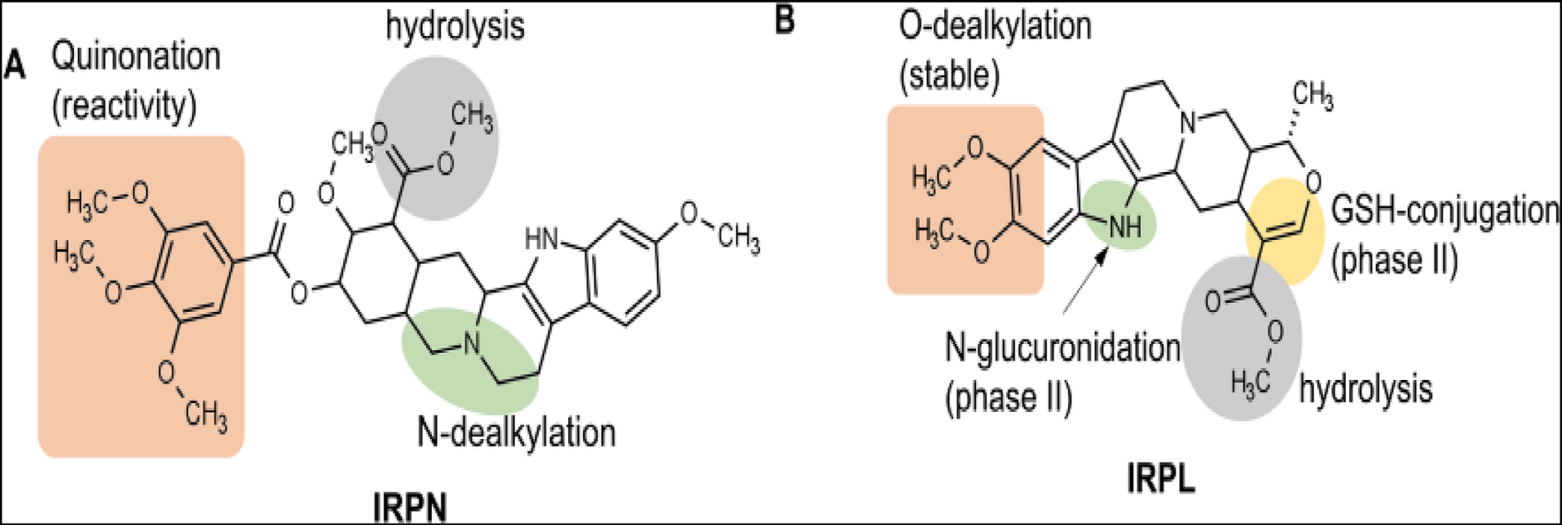
Site of Phase I (CYP450‐dependent oxidation) and Phase II (conjugation reactions) metabolism of compounds (A) IRPN and (B) IRPL.

This predictive analysis evaluates the susceptibility of specific chemical groups and organic functions to metabolism by these CYP450 isoenzymes [32]. Based on this prediction, IRPN was shown to be less resistant to Phase I metabolism compared to its analogue IRPL (Figure 9). Notably, the trimethoxylated ring of IRPN is highly susceptible to quinonation via resonance (Figure 9A), particularly in the conjugated system adjacent to the ester group, leading to the formation of an unstable and reactive metabolite (Figure 9B) [33].

In addition, IRPN presented a hydrolysis site dependent on esterases, as well as a site of *N*‐dealkylation within the fused heterocyclic substructure, both mediated by major CYP450 isoforms (2C9, 2D6, and 3A4) [32]. In contrast, IRPL appears to be more susceptible to Phase II metabolism, including glutathione (GSH) conjugation at the unsaturation site within the oxygenated ring, which reduces its reactivity toward proteins and DNA (Figure 9B) [35]. Moreover, IRPL contains an NH group capable of glucuronidation by the enzyme UDP‐glucuronosyltransferase (UGT). The compound also forms low‐reactivity metabolites during Phase I metabolism (CYP450‐dependent), including *O*‐dealkylation of methoxy groups on the benzene ring of the indole substructure and a hydrolysis site at the ester group, mediated by hepatic esterases (Figure 9B).

Thus, the estimated CLPlasma values of 6.619 mL/min/kg for the IRPN ligand and 9.304 mL/min/kg for the IRPL ligand indicate that metabolic processes do not significantly affect the plasma clearance of these compounds, resulting in favorable oral bioavailability in both cases (Table 5).

**TABLE 5:** HLM stability descriptors expressed as CL and *t*_1/2_ for IRPN and IRPL derivatives predicted using ADMETlab and ADMETboost tools.

Compound	CLHepa (µL/min/10^6^ cells)	CLMicro (mL/min/g	CLPlasma (mL/min/kg)	*t*_1/2_ (h)
IRPN	43.48	58.43	6.619	0.84
IRPL	44.43	53.12	9.304	1.87

Abbreviations: CL, hepatic clearance; *t*_1/2_, half‐life time.

However, the estimated CLMicro value of 56.12 mL/min/g suggests that IRPL tends to be metabolically more stable in the HLM system compared to the IRPN derivative (Table 5) [32]. This prediction is consistent with the estimated half‐life (*t*_1/2_) of 1.87 h for the IRPL compound, which is higher than that of the IRPN derivative (Table 5).

## 3 Conclusions

The assays conducted in adult zebrafish demonstrated that the IRPN and IRPL samples did not exhibit toxicity at the tested doses. Moreover, both compounds showed anxiolytic activity at doses of 4 and 20 mg/kg, with a mechanism of action mediated by the serotonergic pathway.

Molecular docking simulations revealed that the IRPL ligand may act as an antagonist of serotonergic receptors (5‐HT3AR) involved in anxiolytic pathways, primarily through interactions with key receptor modulation residues, including the aromatic residues Trp63 and Trp156. Supporting these findings, DMPK descriptors indicated that IRPL possesses a more favorable pharmacokinetic profile for CNS permeability compared to IRPN, as well as greater metabolic stability, making it a promising drug candidate for the treatment of anxiety via the serotonergic pathway.

## 4 Experimental Section

### 4.1 Drugs and Reagents

In this study, the substances used were fluoxetine (FLX; Sandoz), DZP (Neo Química), dimethyl sulfoxide (DMSO; 3%; Dynamic), and GRAN. The indole alkaloids used in this study were IRPN and IRPL, whose extraction and isolation process from *R. ligustrina*, as well as structural elucidation, have already been carried out by the research group and are documented in the literature [4].

### 4.2 Zebrafish

Zebrafish aged between 90 and 120 days (0.4 ± 0.1 g), both males and females, were obtained from a commercial supplier in Fortaleza, CE. The animals were divided into groups of 50 and acclimated in glass aquariums (30 × 15 × 20 cm) filled with dechlorinated water (ProtecPlus), equipped with air pumps and submerged filters. The water temperature was maintained at 25°C, pH at 7.0, and a light–dark cycle of 10–14 h was established. The fish were fed spirulina feed 24 h before the experiments. At the end of the experiments, the animals were euthanized by immersion in cold water (2°C–4°C) for 10 min until opercular movement ceased. All bioassays conducted were approved by the Animal Use Ethics Committee (CEUA) of the State University of Ceará (protocol 04983945/2021).

### 4.3 Acute Toxicity

Acute toxicity tests were conducted on adult zebrafish according to the guidelines of the Organization for Economic Cooperation and Development (OECD) [37], aiming to determine the lethal dose capable of killing 50% of the animals (LD_50_) in 96 h. Each of the samples, IRPN and IRPL, was administered at doses of 4, 12, or 20 mg/kg via intraperitoneal injection (ip; 20 µL), with each treatment group consisting of six fish. The negative control group received DMSO (3%; 20 µL, ip), and the positive control group received DZP (4 mg/kg; 20 µL, ip). The number of dead fish was recorded in each group every 24 h to determine the LD_50_ [38].

### 4.4 Open Field Test

The open field test was used to evaluate the influence of the samples on the locomotor activity of zebrafish. Each of the samples, IRPN and IRPL, was administered at doses of 4, 12, or 20 mg/kg via intraperitoneal injection (ip; 20 µL), with each experimental group consisting of six fish. The negative control group received DMSO (3%; 20 µL, ip), and the positive control group received DZP (4 mg/kg; 20 µL, ip). Thirty minutes after administration, the fish were individually placed in Petri dishes (10 × 15 cm), divided into four quadrants and filled with the same water from the aquarium. Locomotor activity was assessed for 5 min by counting the number of line crossings (LC) [39].

### 4.5 Anxiolytic Evaluation

The test was conducted in a glass aquarium (30 cm × 15 cm × 20 cm) divided into two zones—light and dark—and filled with dechlorinated tap water to a height of 3 cm. This environment simulates a novel and stressful situation, different from the standard aquarium, and is capable of inducing anxiety‐like behaviors. Each sample, IRPN and IRPL, was administered at doses of 4, 12, or 20 mg/kg via intraperitoneal injection (ip; 20 µL), with experimental groups consisting of six fish. The negative control group received DMSO (3%; 20 µL, ip), while the positive control group received DZP (4 mg/kg; 20 µL, ip). Thirty minutes after administration, the fish were individually placed in the light zone of the aquarium, and the anxiolytic effect was assessed based on the TSLZ over a 5‐min period. At the end of the test, the optimal dose for each sample was determined based on a comparison between the anxiolytic performance and the observed toxicity profile [40].

### 4.6 Serotonergic Neuromodulation Assessment

Groups of six fish were pre‐treated with the antagonist GRAN (5‐HT, 20 mg/kg, orally), and after 30 min, they received the optimal dose of the sample that exhibited anxiolytic effects. The negative control group received DMSO (3%; 20 µL, ip), while the positive control group was treated with FLX (0.05 mg/kg, 10 µL, ip), used as an agonist of the 5‐HT receptor. Subsequently, the groups were subjected to the light/dark test, as described in the previous section [41].

### 4.7 Statistical Analysis

The results were presented as mean ± standard error of the mean (SEM) for each group of six fish. After verifying the normality of distribution and homogeneity of the data, comparisons between groups were performed using one‐way analysis of variance (one‐way ANOVA) for parametric data, followed by Tukey's test. For nonparametric data, the Kruskal–Wallis test was applied, followed by Dunn's test. In experiments involving antagonists, two‐way (ANOVA) was used, also followed by Tukey's test. Statistical analyses were performed using GraphPad Prism software version 10.3.1, adopting a significance level of 5% (*p* < 0.05).

### 4.8 Molecular Docking

To evaluate the theoretical mechanism of the anxiolytic action of the IRPN and IRPL derivatives, molecular docking simulations were performed using the serotonin receptor type 3A (5‐HT3AR) as the target. The Cryo‐EM structure of 5‐HT3A receptor in presence of granisetron, in its characterized conformational space and pdb format, was retrieved from the RCSB Protein Data Bank server (https://www.rcsb.org/), deposited under PDB ID code 6NP0. This structure is classified as a membrane receptor from the organism *Mus musculus* and was characterized by electron microscopy at a resolution of 2.92 Å [24].

The structure was prepared by removing water residues and adding polar hydrogens and Gasteiger charges using the AutoDockTools software (https://autodocksuite.scripps.edu/adt/). The grid box was defined to cover the entire conformational space of the receptor, centered at coordinates 159.620 (*x*), 159.710 (*y*), and 161.073 (*z*), with dimensions of 94 Å × 80 Å × 126 Å, based on the methodology described by Mendes et al. [34].

The AutoDock Vina software (https://vina.scripps.edu/) was configured to perform 50 independent simulations with 20 poses each, using the Lamarckian genetic algorithm (LGA). The best pose was selected based on the alignment of three parameters: (i) RMSD less than 2.0 Å, (ii) binding affinity energy lower than −6.0 kcal/mol, and (iii) ligand–receptor interactions with amino acid residues of the active/catalytic site [42].

### 4.9 NMA‐Based MD Simulation

NMA of the 5‐HT3AR receptor (PDB ID: 6np0) in complex with the ligands IRPN and IRPL was conducted using the iMODS server (https://imods.iqf.csic.es/) [25] to assess the structural flexibility and stability of the protein–ligand complex (ligand–protein complex from molecular docking simulations). The PDB format of the complex was loaded into iMODS, where NMA was performed to evaluate the conformational MD of the complex. The results, including RMSD of alpha carbon (Cα), deformability, and B‐factor predictions, were analyzed to gain insights into the stability and functional movements of the complex. Morphing analysis, when applied to NMA, considers conformational differences between two complexes, including: the ligand–protein complex from molecular docking simulations is the first component, and the initial structure from PDB 6np0 is the second component. The stability of the complex is indicated by the relationships between RMSD Cα and iteration index [25, 26].

Finally, the protein‐binding energy prediction (PRODIGY) tool was used to determine the free binding energy (Δ*G*_binding_) of the three‐dimensional structure of the ligand–protein complex from the output file of the MD simulations (wenmr.science.uu.nl/prodigy/) [43, 44].

### 4.10 In Silico DMPK Studies

To investigate the DMPK properties of the IRPN and IRPL derivatives, descriptors for PAMPA were predicted using the ADMETlab (https://admetlab3.scbdd.com/) and ADMETboost (https://ai‐druglab.smu.edu/admet) platforms, in combination with physicochemical properties analyzed through the quantitative estimate of drug likeness using the CNS MPO algorithm, developed by Pfizer Inc., as shown in Equation (1):

(1) $$d=\sum_{i=1}^{N}w_{k}T_{k}x_{k}^{0}$$

where *w* represents the individual weight factor of each physicochemical property *k* within the threshold functions (*T*(*x*)) defined by: lipophilicity (log *P*) ≤ 3, solubility at physiological pH (log *D*) ≤ 2, molecular weight (MW) ≤ 360 g/mol, TPSA between 40 and 90, H‐bond donors ≤ 1, and basic p*K*_a_ ≤ 8. The summation yields a score ranging from 0 to 6 (*N* = 6 properties), where compounds with CNS MPO > 4.0 are considered to have greater safety for activity in the CNS, due to permeability across the blood–brain barrier (BBB) [45].

The results were correlated with predicted descriptors of apparent permeability (*P*_app_) in the Madin‐Darby canine kidney (MDCK) cell model, efflux potential (*P*_eff_), BBB permeability, and fraction absorbed in the gastrointestinal tract [46].

The metabolic stability of the compounds was estimated through prediction of metabolic sites using the Xenosite server (https://xenosite.org/), where alignment between sensitivity and specificity of chemical groups was used to identify potential oxidation sites (Phase I), dependent on oxidative metabolism by cytochrome P450 (CYP450) isoenzymes, and conjugation sites, dependent on Phase II metabolic enzymes. The structural analysis was related to descriptors of clearance (CL) in the HLM system and half‐life, using the ADMETlab and ADMETboost platforms.

## Author Contributions

**Nádia Aguiar Portela Pinheiro**: investigation, writing, review, editing. **Ivana Carneiro Romão**: supervision, formal analysis, software. **Amanda Maria Barros Alves**: supervision, formal analysis, software. **Sônia Maria Costa Siqueira**: original draft writing, manuscript review. **Jane Eire Silva Alencar de Menezes**: administration, project writing. **Emmanuel Silva Marinho**: software, validation. **Maria Kueirislene Amâncio Ferreira**: software, validation. **Márcia Machado Marinho**: software, validation. **Herbert de Sousa Magalhães**: extraction, isolation, characterization of samples. **Andreia Ferreira de Castro Gomes**: original draft writing, manuscript review. **Otília Deusdênia Loiola Pessoa**: extraction, isolation, characterization of samples. **Hélcio Silva dos Santos**: administration, project writing

## Conflicts of Interest

The authors declare no conflicts of interest.

## Data Availability

The data that support the findings of this study are available from the corresponding author upon reasonable request.
